# Better clinical outcome with direct oral anticoagulants in hospitalized heart failure patients with atrial fibrillation

**DOI:** 10.1186/s12872-018-0746-z

**Published:** 2018-01-25

**Authors:** Akiomi Yoshihisa, Yu Sato, Takamasa Sato, Satoshi Suzuki, Masayoshi Oikawa, Yasuchika Takeishi

**Affiliations:** Department of Cardiovascular Medicine, Fukushima Medical University, 1 Hikarigaoka, Fukushima, 960-1295 Japan

**Keywords:** Heart failure, Atrial fibrillation, Anticoagulant therapy, Direct oral anticoagulants, Vitamin K antagonists, Mortality

## Abstract

**Background:**

Atrial fibrillation (AF) is common in patients with heart failure and is associated with higher mortality. Although previous studies have reported that direct oral anticoagulants (DOACs) reduce the risk of cardiovascular events in out-patients with AF, it remains unclear whether DOACs reduce mortality in hospitalized heart failure (HHF) patients with AF. Therefore, we examined the impact of DOACs on mortality in this group of patients.

**Methods:**

Consecutive 497 HHF patients with AF were retrospectively registered and divided into three groups on the basis of the presence of anticoagulant therapy: non-anticoagulant group (Non, *n* = 90), Vit K antagonists (VKAs) group (*n* = 257) and DOACs group (*n* = 150). We followed up all the patients for mortality.

**Results:**

In the Kaplan-Meier analysis (mean follow-up of 1093 days), all-cause mortality was significantly lower in the VKAs and DOACs groups than in the Non group (31.1% and 15.3% vs. 43.3%, log-rank *P* < 0.001). In the multivariable Cox proportional hazard analysis after adjusting for other potential confounding factors, usage of DOACs and VKAs were independently associated with lower mortality in HHF patients AF (DOACs, HR 0.356, *P* = 0.001; VKAs, HR 0.472, *P* = 0.002). Furthermore, the propensity-matched 1:1 cohort was assessed based on the propensity score (DOACs, *n* = 114 and VKAs, n = 114). All-cause mortality was significantly lower in the DOACs group than in the VKAs group in the post-matched cohort (12.3% vs. 35.1%, log-rank *P* = 0.038). In the Cox proportional hazard analysis, the use of DOACs was associated with lower mortality in the post-matched cohort (HR 0.526, *P* = 0.041).

**Conclusion:**

Appropriate use of anticoagulants in HHF patients with AF is important, and DOACs potentially improve all-cause mortality in such patients.

**Electronic supplementary material:**

The online version of this article (10.1186/s12872-018-0746-z) contains supplementary material, which is available to authorized users.

## Background

Heart failure (HF) is a systemic disease with a devastating prognosis, which affects many organ systems, including the cardiovascular system. In HF patients, atrial fibrillation (AF) is a frequent co-morbidity and its prevalence is related to the severity of the clinical status of patients [1]. HF and AF share common risk factors and clinical backgrounds (e.g. inflammation and fibrosis), and the occurrence of either of them may induce the onset of a vicious circle which, in turn, facilitates the manifestation of the other [2–4]. AF subsequently causes stroke and/or HF [5]. On the other hand, the incidence of AF increases with severity of HF [4], and AF in HF patients is strongly associated with all-cause mortality including sudden cardiac death [6] and bleeding, rather than stroke or embolism [7–9]. Hence, all-cause mortality is the most meaningful endpoint in such patients.

Anticoagulant therapy is prescribed in consideration of balance of efficacy for embolic risk (e.g. CHADS score, CHA_2_DS_2_-VASc score) and safety for bleeding risk (e.g. HAS-BLED score) in AF patients [10]. Direct oral anticoagulants (DOACs) have the potential to reduce the burden of stroke as effective, safe, and more convenient alternatives to vitamin K antagonists (VKAs), such as warfarin [11–13]. A recent meta-analysis of randomized controlled studies revealed that DOACs significantly reduce the risk of stroke or systemic embolic events by 19% and hemorrhagic stroke by 51% compared with VKAs, but increased the risk of gastrointestinal hemorrhage by 25% in out-patients with AF [12]. On the other hand, a few post-hoc analyses limited to AF out-patients with HF revealed that 1) DOACs were similarly effective or safer (less intracranial hemorrhage) compared to VKA in AF out-patients with HF compared with those without HF, and that 2) DOACs have not yet improved all-cause mortality of AF out-patients with HF [7–9, 14]. However, there were several problems in the previous studies [7–9, 14]: 1) out-patients with reduced left ventricular ejection fraction and no symptoms of HF were included in previous studies, [7–9, 14] and hospitalized heart failure (HHF) patients had higher accurate diagnosis of HF and risk of mortality than out-patients [15–17], 2) important factors for HF, such as Framingham criteria, etiology, presence of anemia and hyponatremia, natriuretic peptide, medications of HF, were partly absent or were not considered in these post-hoc analyses [7–9, 14], 3) anticoagulant therapy could not be used in all the patients with HF and AF in a real world setting [18] because of certain contraindications for anticoagulants (higher HAS-BLED score and presence of anemia) [18]. Additionally, studies on symptomatic HHF patient coexistence with AF have not been previously reported. The association between DOACs and all-cause mortality in HHF patients with AF is still unclear and controversial. Therefore, we examined the impact of anticoagulant therapy on all-cause mortality in symptomatic HHF patients with AF based on a retrospective study, using propensity score (PS) analyses to reduce selection bias, and taking into consideration the patients’ clinical backgrounds, including CHA_2_DS_2_-Vasc and HAS-BLED scores, other co-morbidities and pharmacotherapies.

## Methods

### Subjects and study protocol

**Fig. 1:**
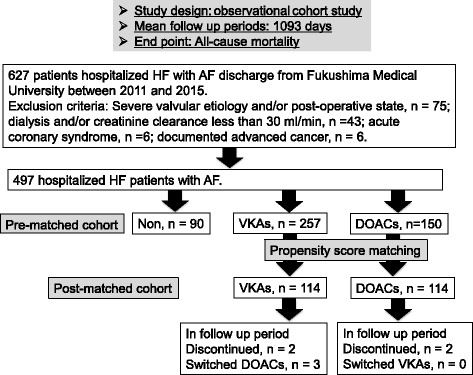
Patient flow chart

This was a observational study analyzed using PS methods in which we enrolled consecutive HHF patients with AF, who were hospitalized with decompensated HF defined based on the Framingham criteria [19]., and discharged from Fukushima Medical University Hospital between 2011 and 2015. AF was identified by an electrocardiogram performed during hospitalization and/or medical records including past history. The patient flow chart is shown in Fig. 1. In HHF patients with AF (*n* = 627), patients with severe valvular etiology and/or post-operative state (*n* = 75), dialysis and/or creatinine clearance less than 30 ml/min (*n* = 43), acute coronary syndrome (*n* = 6), and documented advanced cancer (*n* = 6) were excluded, and 497 HHF patients with AF were finally enrolled. Patients were divided into three groups on the basis of the use of VKAs and DOACs at hospital discharge: non-anticoagulant group (Non, *n* = 90), VKAs group (warfarin, *n* = 257) and DOACs group (*n* = 150). The DOACs used in this study were as follows: apixaban (*n* = 52, 34.7%), rivaroxaban (*n* = 35, 23.3%), edoxaban (*n* = 33, 22.0%) and dabigatran (*n* = 30, 20.0%). We compared the clinical features and laboratory data that were recorded at hospital discharge. The CHADS score, CHA_2_DS_2_-VASc score, HAS-BLED score, and time in therapeutic range (TTR) of VKAs were measured as previously reported [10]. The left ventricular ejection fraction (LVEF) was calculated using Simpson’s method, and recordings were performed on ultrasound systems (ACUSON Sequoia, Siemens Medical Solutions USA, Inc., Mountain View, CA, USA). Preserved LVEF was defined as more than LVEF 50%. All patients were followed up until 2017 for all-cause mortality. Cardiac death was defined by experienced cardiologists as death due to worsening HF in accordance with the Framingham criteria [19], due to ventricular fibrillation determined by electrocardiogram or implantable devices, and acute myocardial infarction. Survival time was calculated from the day of discharge to the date of death or last follow-up. Status and dates of deaths were checked from medical recoreds of the patients. If these data were unavailable, status was examined by a telephone call to the patient’s referring hospital physicians. The end-point classification committee, comprising two experienced cardiologists who were not study investigators, reviewed the data. We could follow up all the patients.

Written informed consent was obtained from each subject, and the study protocol was approved by the ethics committee of Fukushima Medical University (No. 823). The investigation conforms with the principles outlined in the Declaration of Helsinki. Reporting of the study conforms to STROBE along with references to STROBE and the broader EQUATOR guidelines [20].

### Statistical analysis

Categorical variables are expressed as numbers and percentages, and the chi-square test was used for their comparisons. Normally distributed data are presented as mean ± SD, and non-normally distributed data are presented as median and interquartile range, or log-transformed. Data among the three groups were compared using analysis of variance followed by Tukey’s post-hoc test. The Kaplan-Meier method was used for presenting the mortality, and the log-rank test was performed.

To eliminate imbalances in the measurement of baseline characteristics because of selection bias, we used multiple approaches, including multiple Cox regression analysis in the pre-matched cohort (*n* = 497) and PS matching in the post-matched cohort (*n* = 228). To prepare for potential confounding in the Cox regression analyses, we considered the following clinical factors, which are known to affect the mortality in HF patients: age, sex, B-type natriuretic peptide (BNP), presence of New York Heart Association functional class III or IV, ischemic etiology, preserved LVEF, hypertension, diabetes, CKD, anemia, hyponatremia (as sodium less than 135 mEq/l), and oral administration of renin-angiotensin-aldosterone system inhibitors, β-blockers, diuretics, inotropic agents, and anticoagulants. These factors, which predicting mortality with value of *P* < 0.05, were selected in the final adjusted model.

Furthermore, the PS for treatment with DOAC was estimated for each patient by logistic regression with the following variables, which are important considerable factors to prescribe DOACs: age, body weight, CHA_2_DS_2_-Vasc score, HAS-BLED score and creatinine clearance. The PS is the propensity from 0 to 1 to receive treatment, and is used to adjust potential selection bias and confounding factors between the groups with or without treatment in the observational studies [21]. We used the PS to match patients who were administered DOAC and those who were administered VKA, using 1:1 nearest neighbor matching algorithm and a 0.2 caliper width of the standard deviation of the PS logit with caliper as of 0.03 [22]. The PS-matched datasets were compared using a pairwise analysis [23], and the post-matched cohort (DOACs, *n* = 114 and VKAs, *n* = 114) was defined. To assess potential heterogeneity of the effect of DOAC on mortality, we conducted subgroup analyses in the post-matched cohort (*n* = 228). We tested for first order interactions using multivariable Cox proportional hazard models by entering interaction terms between DOAC use and the subgroup variables. Missing values were handled by estimating one logistic regression model for each pattern of missing values. A *P* value of < 0.05 was considered significant for all comparisons. These analyses were performed using a statistical software package (SPSS ver. 24.0, IBM, Armonk, NY, USA).

## Results

The clinical features of the study subjects are presented in Table 1. The Non group had the highest age, HAS-BLED score, prevalence of paroxysmal AF, female gender and anemia. It seems that this background is associated with abandonment of anticoagulants. The VKAs group had the highest prevalence of male gender and hypertension, and highest usage of β-blockers, diuretics, and inotropic agents. In addition, TTR was 70% in VKAs group (data not shown in Table 1). The DOACs group had the lowest prevalence of anemia. In contrast, CHADS_2_ and CHA_2_DS_2_-Vasc scores, prevalence of NYHA class III or IV, preserved LVEF, other co-morbidities, BNP, C-reactive protein and sodium did not significantly differ among the three groups.

**Table 1:** Comparisons of clinical features (*n* = 497)

	Non (*n* = 90)	VKAs (*n* = 257)	DOACs (*n* = 150)	*P* value
Age (years)	74.4 ± 12.0	69.9 ± 11.8**	70.4 ± 12.9*	0.010
CHADS_2_ score	3.2 ± 1.3	3.1 ± 1.2	2.9 ± 1.3	0.286
CHA_2_DS_2_-Vasc score	4.8 ± 1.6	4.4 ± 1.7	4.3 ± 1.8	0.074
HAS-BLED score	3.4 ± 1.3	2.8 ± 1.2**	2.7 ± 1.4*	0.006
Paroxysmal af (n, %)	44 (48.9)	89 (34.6)	52 (34.7)	0.041
Male gender (n, %)	44 (48.9)	175 (68.1)	89 (59.3)	0.004
Body mass index (kg/cm^2^)	22.7 ± 4.9	23.3 ± 4.0	23.5 ± 3.9	0.404
Systolic BP (mmHg)	130.8 ± 33.1	123.8 ± 28.8	132.3 ± 36.3	0.021
Diastolic BP (mmHg)	72.8 ± 19.2	72.6 ± 18.6	76.0 ± 24.3	0.265
Heart rate (bpm)	89.7 ± 35.0	84.1 ± 29.1	85.5 ± 35.8	0.371
NYHA class III or IV (n, %)	6 (6.7)	8 (3.1)	4 (2.7)	0.226
Preserved LVEF (n, %)	41 (45.6)	126 (49.0)	67 (44.7)	0.662
Etiology				0.130
Ischemic (n, %)	26 (28.9)	68 (26.5)	43 (28.7)	
Cardiomyopathy (n, %)	23 (25.6)	68 (26.5)	36 (24.0)	
Valvular (n, %)	19 (21.1)	70 (27.2)	25 (16.7)	
Others (n, %)	22 (24.4)	51 (19.8)	46 (30.7)	
Co-morbidity				
Hypertension (n, %)	67 (74.4)	202 (78.6)	98 (65.3)	0.013
Diabetes (n, %)	36 (40.0)	103 (40.1)	53 (35.3)	0.611
Dyslipidemia (n, %)	66 (73.3)	185 (72.0)	97 (64.7)	0.224
CKD (n, %)	49 (54.4)	163 (63.4)	85 (56.7)	0.213
Anemia (n, %)	58 (64.4)	144 (56.0)	70 (46.7)	0.023
Stroke (n, %)	23 (25.6)	65 (25.3)	32 (21.3)	0.628
Medications				
RAS inhibitors (n, %)	61 (67.8)	206 (80.2)	115 (76.7)	0.057
β-blockers (n, %)	63 (70.0)	219 (85.2)	116 (77.3)	0.005
Diuretics (n, %)	55 (61.1)	214 (83.3)	103 (68.7)	< 0.001
Inotropic agents (n, %)	9 (10.0)	52 (20.2)	7 (4.7)	< 0.001
Antiplatelet agents (n, %)	43 (47.8)	108 (42.0)	62 (41.3)	0.576
Laboratory data				
BNP (pg/ml) §	332.8 (140.3–656.1)	284.1 (157.4–625.9)	270.8 (101.8–552.2)	0.494
C-reactive protein (mg/dl) §	0.13 (0.05–0.53)	0.07 (0.03–0.18)	0.08 (0.04–0.25)	0.104
Sodium (mEq/l)	138.6 ± 5.1	138.7 ± 4.0	139.2 ± 4.3	0.561

*Af* atrial fibrillation, *BP* blood pressure, *NYHA* New York Heart Association, *LVEF* left ventricular ejection fraction, *CKD* chronic kidney disease, *RAS* rennin-angiotensin-aldosterone system; *BNP* B-type natriuretic peptide

*P < 0.05 and **P < 0.01 vs. Non group

§Data are presented as median (interquartile range)

**Fig. 2:**
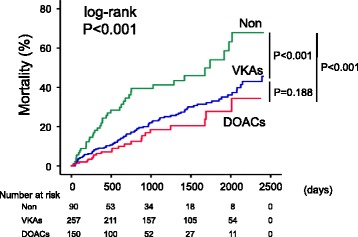
All-cause mortality: pre-matched cohort. Kaplan-Meier analyses for all-cause mortality among the three groups (Non group, *n* = 90; VKAs group, *n* = 257; DOACs group, *n* = 150) in the pre-matched cohort (*n* = 497);

In the follow-up period (mean of 1093 days), 70 cardiac deaths (worsened HF *n* = 42, ventricular fibrillation *n* = 22 and acute coronary syndrome *n* = 6) and 72 non-cardiac deaths (stroke *n* = 10, gastrointestinal hemorrhage *n* = 7, aneurysms *n* = 5, trauma *n* = 4, pneumonia/respiratory failure/hemorrhage *n* = 18, cancer *n* = 12, infection/disseminated intravascular coagulation *n* = 5, systemic embolism, *n* = 3, renal failure *n* = 3, others *n* = 5) occurred. As shown in Fig. 2, all-cause mortality was significantly lower in the DOACs and VKAs groups than in the Non group in the pre-matched cohort (Fig. 2; *P* < 0.001). In the Cox proportional hazard analysis after adjusting for potential confounding factors (Table 2), the usage of DOACs and VKAs was independently associated with lower mortality in HHF patients with AF (DOACs, HR 0.356, *P* = 0.001; VKAs, HR 0.472, *P* = 0.002).

**Table 2:** Cox proportional hazard model of all-cause mortality (event = 142/ *n* = 497)

Risk factor	Univariable			Multivariable		
	HR	95% Cl	*P* value	HR	95% Cl	P value
Age	1.045	1.028–1.063	< 0.001	1.025	1.007–1.044	0.007
Male sex	1.044	0.741–1.472	0.805			
NYHA class III or IV	4.662	2.718–7.995	< 0.001	1.561	0.826–2.949	0.170
Ischemic etiology	1.031	0.682–1.560	0.885			
Preserved LVEF	0.770	0.552–1.073	0.122			
Hypertension	1.089	0.719–1.651	0.687			
Diabetes	1.331	0.957–1.851	0.089			
Chronic kidney disease	1.842	1.274–2.664	0.001	1.352	0.899–2.032	0.147
Anemia	2.456	1.688–3.573	< 0.001	1.849	1.214–2.816	0.004
Hyponatremia	2.089	1.346–3.241	0.001	1.406	0.890–2.222	0.144
Log BNP	3.541	2.394–5.238	< 0.001	2.970	1.903–4.635	< 0.001
RAS inhibitors	0.632	0.433–0.921	0.017	0.750	0.492–1.141	0.179
β-blockers	0.636	0.437–0.927	0.018	0.657	0.426–1.014	0.058
Diuretics	2.290	1.379–3.802	0.001	2.008	1.137–3.546	0.016
Inotropic agents	2.026	1.377–2.982	< 0.001	2.126	1.366–2.308	0.001
Anticoagulants: Non	Ref			Ref		
VKAs	0.493	0.335–0.725	< 0.001	0.472	0.296–0.750	0.002
DOACs	0.352	0.210–0.590	< 0.001	0.356	0.199–0.638	0.001

*NYHA* New York Heart Association, *LVEF* left ventricular ejection fraction, *BNP* B-type natriuretic peptide, *RAS* renin-angiotensin-aldosterone system, *VKAs* vitamin K antagonists, *DOACs* direct oral anti-coagulants

**Fig. 3:**
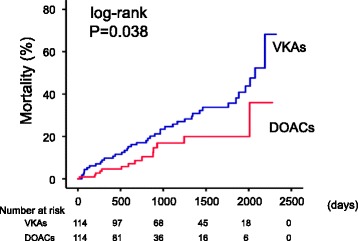
All-cause mortality: post-matched cohort. Kaplan-Meier analyses for all-cause mortality between the groups (VKAs group, *n* = 114 and DOACs group, *n* = 114) in the post-matched cohort (*n* = 228)

In addition, in the post-matched cohort, mortality was significantly lower in the DOACs group than in the VKAs group (Fig. 3; *P* = 0.038). The clinical features of DOACs group and VKAs group in the post-matched cohort are summarized in Additional file 1: Table S1. Interactions between the DOACs group and clinically relevant variables were modeled with the Cox regression analysis, as shown in Table 3, for all-cause mortality in the post-matched cohort (*n* = 228). In the Cox proportional hazard analysis (Table 3), the usage of DOACs was associated with lower mortality in the post-matched cohort (HR 0.526, 95% CI 0.284–0.974, *P* = 0.041). There were no interactions between DOACs use and other important variables (e.g. CKD, anemia, LVEF, other pharmacotherapies) that affected mortality in all subgroups.

**Table 3:** Subgroup analysis for all-cause mortality: DOACs vs. VKAs use in post matched cohort

Factor	Subgroup	n	HR	95% CI	P value	Interaction P value
DOACs vs. VKAs	Total	228	0.526	0.284–0.974	0.041	–
Age	≥ 70	126	0.604	0.299–1.219	0.159	0.537
	< 70	102	0.331	0.091–1.206	0.094	
Sex	Male	159	0.569	0.287–1.127	0.106	0.809
	Female	69	0.539	0.108–2.685	0.451	
NYHA class	I, II	222	0.561	0.301-1.044	0.068	0.978
	III, IV	6	0.024	0.000-397.4	0.540	
BNP	>median	114	0.341	0.115-1.012	0.053	0.168
	<median	114	0.690	0.319-1.489	0.344	
LVEF	Reduced	127	0.676	0.312–1.465	0.321	0.245
	Preserved	101	0.366	0.132–1.014	0.053	
Ischemic etiology	Present	63	0.926	0.319–2.002	0.466	0.269
	Absent	165	0.435	0.207–0.915	0.028	
Diabetes	Present	92	0.693	0.302–1.594	0.388	0.364
	Absent	136	0.401	0.159–1.011	0.053	
CKD	Present	131	0.598	0.302–1.185	0.141	0.994
	Absent	97	0.667	0.152–2.931	0.591	
Anemia	Present	106	0.474	0.215–1.044	0.064	0.498
	Absent	122	0.729	0.251–2.112	0.560	
RAS inhibitors	Present	188	0.664	0.336–1.313	0.239	0.171
	Absent	40	0.187	0.041–0.861	0.031	
β-blockers	Present	186	0.564	0.288–1.106	0.096	0.389
	Absent	42	0.293	0.061–1.394	0.123	
Diuretics	Present	166	0.630	0.333–1.191	0.155	0.475
	Absent	62	0.452	0.041–4.992	0.517	
Antiplatelet agents	Present	97	0.863	0.386–1.931	0.720	0.117
	Absent	131	0.295	0.109–0.796	0.016	

*DOACs* direct oral anticoagulants, *VKAs* Vit K antagonists, *NYHA* New York Heart Association, *BNP* B-type natriuretic peptide, *LVEF* left ventricular ejection fraction, *CKD* chronic kidney disease, *RAS* rennin-angiotensin-aldosterone system

## Discussion

To the best of our knowledge, the present study is the first to show the association between DOACs and lower all-cause mortality in HHF patients with AF based on a real world observational study using multiple Cox regression and PS analyses, considering clinical backgrounds, including CHA_2_DS_2_-Vasc and HAS-BLED scores, other co-morbidities, and pharmacotherapies.

To improve the prognosis of HHF patients with AF, prevention of stroke and systemic embolism, as well as avoidance of major bleeding, may be the therapeutic target. To this point, appropriate use of DOACs is expected to be associated with better prognosis in HHF patients with AF. There are several randomized clinical trials in out-patients with AF regarding efficacy (prevention of stroke and/or systemic embolism) and safety (avoidance of intra cranial hemorrhage or gastrointestinal hemorrhage) of DOACs compared with VKAs. Firstly, dabigatran tended to lower all-cause mortality (RR 0.88, 95% CI 0.77–1.00) in the RE-LY trial, with enrolled 18,113 out-patients with AF (CHADS_2_ score = 2.1, TTR = 67%, HF patients 32%) [24]. In the post-hoc analysis, the relative effects of dabigatran, compared to VKAs, on the occurrence of stroke or systemic embolism and major bleeding were consistent among those with or without HF and those with reduced or preserved LVEF [7]. Secondly, rivaroxaban tended to lower all-cause mortality (RR 0.85, 95% CI 0.70–1.02) in the ROCKET-AF trial, with 14,264 enrolled out-patients with AF (CHADS_2_ score = 3.5, TTR = 58%, HF patients 63.7%) [25]. In the post-hoc analyses, the efficacy of rivaroxaban was similar in AF out-patients with or without HF [9]. Among the AF patients with HF, the efficacy of rivaroxaban was similar, irrespective of NYHA class, CHADS_2_ score, and LVEF [9]. Thirdly, apixaban significantly decreased all-cause mortality (RR 0.89, 95% CI 0.80–0.998) in the ARISTOTLE trial, with 18,201 enrolled out-patients with AF (CHADS_2_ score = 2.1, TTR = 66%, HF patients 35%) [26]. In the post-hoc analyses, apixaban reduced the risk of stroke, systemic embolism or all-cause death, irrespective of the presence of HF and/or reduced LVEF [8]. Fourthly, edoxaban decreased cardiovascular mortality (RR 0.87, 95% CI 0.78–0.96) in the ENGAGE-AF TIMI 48, with 21,105 enrolled out-patients with AF (CHADS_2_ score = 2.8, TTR = 68%, HF patients 58%) [27]. Although these previous post-hoc analyses [7–9] are partially concordant with our results, detailed data of HF, such as Framingham criteria, etiology of HF, natriuretic peptide and other co-morbidities, were unknown unlike in our results. Furthermore, there is no report regarding efficacy of DOACs on mortality in HHF patients with AF.

In previous studies regarding DOACs compared to VKAs in AF out-patients with HF, 57% took ACE inhibitors and 68% took β blockers in the RE-LY trial [7], and 60% took ACE inhibitors and 69% took β blockers in the ROCKET AF trial, in which rivaroxaban tended to decrease all-cause mortality (RR 0.93, 95% CI 0.82–1.07) [9]. In the ARISTOTLE trial, 71% took ACE inhibitors and 71% took β blockers; however, apixaban did not decrease mortality (reduced LVEF, RR 0.98, 95% CI 0.79–1.21; preserved LVEF, RR 0.89, 95% CI 0.69–1.13) [8]. Our study subjects were HHF, and had a relatively higher CHADS_2_ score of 3.1, TTR of 70%, and higher usage of RAS inhibitors (76.9%) and β blockers (80.1%). In the present study, the use of DOACs was associated with lower all-cause mortality than VKAs and non-anticoagulant in HHF patient with AF. HF is reportedly associated with poor control of VKAs [28]. It has been recently reported that DOACs, compared with VKAs, is associated with lower prevalence of major bleeding [14] and avoidance of anemia, as well as lower occurrence of kidney injury [29], acute coronary syndrome [30] and HF [30]. These backgrounds may be associated with lower all-cause mortality in HHF patients with AF.

### Study strengths and limitations

Our study has several strengths, and differs from previous studies [7–9, 14]. For instance, the present study is the first to show the effect of DOACs on reducing all-cause mortality in HHF patients with AF considering the clinical background, including CHA_2_DS_2_-Vasc and HAS-BLED scores, other co-morbidities, and pharmacotherapies. In addition, the diagnosis of HF and causes of death were accurately made by our experienced cardiologists, based on the Framingham criteria. Furthermore, there were no patients who dropped out.

There are some potential limitations. Firstly, our study was a observational study at a single institution, and thus the numbers of subjects were relatively small. Although the PS analyses are useful, they are inherently limited by the number and accuracy of the variables evaluated. Importantly, we cannot rule out residual confounding from unknown or unmeasured variables. There might be potential bias and although we analyzed present data using multivariate Cox proportional hazard regression analyses and PS analyses to minimize selection bias under consideration of multiple confounding factors, the effects of the differences in clinical background between the groups might not be completely adjusted. Secondly, we have assessed this study using only variables on hospitalization, without consideration of changes in medical parameters and post-discharge treatment. There might be some cross over in medication during follow up. Thirdly, TTR of VKAs was insufficient; however, TTR in this study was higher than those of previous studies [7–9]. Finally, the differences of each DOAC were not considered in this study. For these reasons, the results of our study should be viewed as preliminary. Hence, further clinical trials for HHF patients with concomitant AF using DOACs are required with a larger population and/or randomization.

## Conclusion

The appropriate use of anticoagulants in HHF patients with AF is important, and DOACs potentially improve all-cause mortality in such patients.

## Additional file

Additional file 1: Table S1. The clinical features of DOACs group and VKAs group in the post-matched cohort are summarized in **Table S1.** (DOC 69 kb)

## Abbreviations

AF: Atrial fibrillation
BNP: B-type natriuretic peptide
CI: Confidence interval
CKD: Chronic kidney disease
DOACs: Direct oral anticoagulants
HF: Heart failure
HHF: Hospitalized heart failure
HR: Hazard ratio
LVEF: Left ventricular ejection fraction
PS: Propensity score
RR: Relative risk
SR: Sinus rhythm
TTR: Time in therapeutic range
VKAs: Vit K antagonists
