# Supplementary material for: Better clinical outcome with direct oral anticoagulants in hospitalized heart failure patients with atrial fibrillation
Source: BMC Cardiovasc Disord. 2018 Jan 25;18:11. doi: 10.1186/s12872-018-0746-z (PMC5784680; doi:10.1186/s12872-018-0746-z)
Supplement: Additional file 1: Table S1. — The clinical features of DOACs group and VKAs group in the post-matched cohort are summarized in Table S1. (DOC 69 kb) [file 12872_2018_746_MOESM1_ESM.doc]

**Supplementary Table 1. Comparisons of clinical features: post matched cohort (n = 228**)

|  | **VKAs**  **(n = 114)** | **DOACs**  **(n = 114)** | ***P* value** |
| --- | --- | --- | --- |
| Age (years) | 70.4 ± 11.3 | 70.5 ± 12.3 | 0.741 |
| CHADS_2_ score | 3.0 ± 1.1 | 3.1 ± 0.9 | 0.613 |
| CHA_2_DS_2_-Vasc score | 4.3 ± 1.5 | 4.3 ± 1.2 | 0.820 |
| HAS-BLED score | 2.9 ± 1.2 | 2.8 ± 1.1 | 0.718 |
| Paroxysmal af (n, %) | 33 (28.9) | 26 (22.8) | 0.290 |
| Male gender (n, %) | 80 (70.2) | 79 (69.3) | 0.885 |
| Body mass index (kg/cm^2^) | 23.7 ± 4.2 | 23.7 ± 3.9 | 0.872 |
| Systolic BP (mmHg) | 125.0 ± 26.3 | 130.1 ± 33.6 | 0.199 |
| Diastolic BP (mmHg) | 74.1 ± 18.2 | 76.3 ± 22.8 | 0.417 |
| Heart rate (bpm) | 80.7 ± 26.0 | 83.2 ± 33.9 | 0.534 |
| NYHA class III or IV (n, %) | 3 (2.6) | 3 (2.6) | 1.000 |
| Preserved LVEF (n, %) | 54 (47.4) | 47 (41.2) | 0.351 |
| Etiology |  |  | 0.373 |
| Ischemic (n, %) | 32 (28.1) | 31 (27.2) |  |
| Cardiomyopathy (n, %) | 39 (34.2) | 35 (30.7) |  |
| Valvular (n, %) | 27 (23.7) | 22 (19.3) |  |
| Others (n, %) | 16 (14.0) | 26 (22.8) |  |
| Co-morbidity |  |  |  |
| Hypertension (n, %) | 81 (71.1) | 73 (64.0) | 0.258 |
| Diabetes (n, %) | 50 (43.9) | 42 (36.8) | 0.280 |
| Dyslipidemia (n, %) | 87 (76.3) | 74 (64.9) | 0.059 |
| CKD (n, %) | 70 (61.4) | 61 (53.5) | 0.228 |
| Anemia (n, %) | 57 (50.0) | 49 (43.0) | 0.288 |
| Stroke (n, %) | 27 (23.7) | 23 (20.2) | 0.522 |
| Medications |  |  |  |
| RAS inhibitors (n, %) | 97 (85.1) | 91 (79.8) | 0.296 |
| β-blockers (n, %) | 95 (83.3) | 91 (79.8) | 0.494 |
| Diuretics (n, %) | 87 (76.3) | 79 (69.3) | 0.234 |
| Inotropic agents (n, %) | 14 (12.3) | 7 (6.1) | 0.109 |
| Antiplatelet agents (n, %) | 53 (46.5) | 44 (38.6) | 0.228 |
| Laboratory data |  |  |  |
| BNP (pg/ml) | 279.0 (145.5–574.2) | 260.5 (105.4–518.5) | 0.464 |
| C-reactive protein (mg/dl) | 0.08 (0.04–0.26) | 0.07 (0.03–0.16) | 0.737 |
| Sodium (mEq/l) | 138.7 ± 3.7 | 139.4 ± 3.9 | 0.152 |

Af, atrial fibrillation; BP, blood pressure; NYHA, New York Heart Association; LVEF, left ventricular ejection fraction; CKD, chronic kidney disease; RAS, rennin-angiotensin-aldosterone system; BNP, B-type natriuretic peptide.

*P<0.05 and **P<0.01 vs. Non group, †P<0.05 and ††P<0.01 vs. VKAs group.

§ Data are presented as median (interquartile range).
